# pH and reduction dual-responsive micelles based on novel polyurethanes with detachable poly(2-ethyl-2-oxazoline) shell for controlled release of doxorubicin

**DOI:** 10.1080/10717544.2019.1580323

**Published:** 2019-03-21

**Authors:** Leran Bu, Hena Zhang, Kang Xu, Baixiang Du, Caihong Zhu, Yuling Li

**Affiliations:** a School of Chemistry & Materials Science, Jiangsu Key Laboratory of Green Synthetic Chemistry for Functional Materials, Jiangsu Normal University, Xuzhou, China;; b Orthopaedic Institute, Medical College, Soochow University, Suzhou, China

**Keywords:** Polyurethane, micelle, dual- responsive, poly(2-ethyl-2-oxazoline), drug delivery

## Abstract

We describe a biodegradable amphiphilic polyurethane (PU) with disulfide bonds in the main chain [PEtOz-*b*-PU(SS)-*b*-PEtOz]. This multi-block PU was synthesized using poly (ε-caprolactone) diol (PCL-SS-PCL) and poly (2-ethyl-2-oxazoline) (PEtOz-OH) as soft segments, and bis (2-isocyanatoethyl) disulfide as the hard segment. Acid-sensitive PEtOz-OH was used as a hydrophilic segment for pH sensitivity. And reduction sensitivity was induced via disulfide bonds incorporated into the hydrophobic poly (ε-caprolactone) segment of the amphiphilic PUs. The system can self-assemble to form micelles responsive to pH and reducing conditions. The properties of the micelle were studied with dynamic light scattering and scanning electron microscopy. Doxorubicin (DOX) was chosen as a model drug. The *in vitro* release studies showed that PEtOz-*b*-PU(SS)-*b*-PEtOz micelle could degrade more rapidly and completely in a reductive and acidic environment [10 mM dl-Dithiothreitol, pH 5.0]. The methyl tetrazolium (MTT) assay and fluorescent microscopy confirmed the cytotoxicity of the DOX-loaded micelles. This work provides a promising dual-responsive drug carrier based on amphiphilic PU to achieve efficient drug delivery.

## Introduction

Anticancer drug delivery systems such as liposomes, micelles, or nanoparticles can overcome some limitations of anticancer drugs including poor solubility in water, high toxicity to normal cells, poor stability in circulation, etc. Although such drug carriers have good biocompatibility, drugs loaded into carrier can be released very slowly. This can result in poor outcomes. Recently, researchers have exploited the features of cancer to design stimuli-responsive nanocarriers for targeted release including in response to reducing conditions or low pH. Drug delivery systems with disulfide (–SS–) bridges can release drugs under reducing conditions (Meng et al., 2009; Huo et al., 2014), and pH-response systems could be constructed by introducing segments that are unstable under acidic conditions (De Leo et al., 2018; Z. Wang et al., 2018). However, single responsive systems have had limited success (Chen et al., 2015) with incomplete release of drug. Thus, polymer nanocarriers sensitive to multiple stimuli might solve this problem (Guan et al., 2017; John et al., 2017).

Polyurethanes (PUs) are an important multi-function material that were first synthesized and commercialized in 1930 (Guelcher, 2008). Since then, PU materials were successfully incorporated into biomaterials. PUs have been widely used in the manufacture of cardiac valves, catheters, artificial blood vessels, artificial limbs, and other implantable devices (Ren et al., 2016; Fu et al., 2017). Biodegradable PUs have also been used for drug delivery and tissue engineering (Zhang et al., 2012; Sulistio et al., 2017). PU offers excellent biocompatibility, perfect biodegradability (Feng et al., 2017; Taraghi et al., 2017), simple synthetic processing, and multifunctional blocks (Ding et al., 2013; Chen et al., 2015; Han et al., 2015). These advantages have caused PU materials to be useful in drug delivery (Cheng et al., 2011; He et al., 2016; Yao et al., 2016).

Drug-loaded PU micelles have been designed to response to pH (Kumari et al., 2010) or reducing conditions (H. Kim et al., 2011; Shao et al., 2013), and enzyme (Cerritelli et al., 2007) environment of tumor cells (Kausar, 2017). Lu et al. (2015) reported multiblock PU micelles with redox reactivity via the introduction of disulfide bonds on PU main chain. These drug-loaded micelles released 74% of the drug in the presence of 10 mM GSH within 12 h. However, nearly 30% of the drug was not released from this micelle. Incomplete release of drug from the polymer micelle is common in other reports (Ding et al., 2012).

He et al. (2014) reported reduction-sensitive multi-block PU micelles with different amounts of disulfide linkages in the polymer backbone. The amount of disulfide bonds was controlled by the excellent molecular tunability of PUs with paclitaxel (PTX) as the model anticancer drug. The PTX-loaded micelle could only release 60% of PTX under reducing conditions within 48 h. Li et al. (2015) optimized the release profiles of redox-sensitive PU based micelles by incorporating different amount of disulfide linkages in hard segment of polymer. Polymers with the largest number of disulfide bonds only released 72% of Doxorubicin (DOX) after 96 h making the drug release process slow and incomplete (John et al., 2015; Yao, et al., 2016). This limits the efficacy or could lead to drug resistance (Perez-Herrero & Fernandez-Medarde, 2015).

Dual responsive and shell-detachable micelles have been developed to release the cargo completely and rapidly (Chen et al., 2017; John et al., 2017). The pH- and redox-active carriers can avoid the accumulation of hydrophobic cores that often leads to incomplete release of drugs in hydrophobic aggregates (Guan et al., 2017; John et al., 2017). Shell-detachable micelles with a disulfide-linked hydrophilic shell and a hydrophobic core were self-assembled via disulfide-linked biodegradable multi-block copolymers. Thus, shell-detachable micelles could detach from the shell material under reducing environments resulting in rapid drug release with destruction of the micellar structure. For example, Wang et al. (2013) reported a novel micelle with detachable PEG corona: the PEG corona of the micelle was detached due to the cleavage of disulfide bonds in the intracellular environment followed by collapse of the micelle. The micelle drastically accelerated drug release at the cytoplasmic GSH level inhibiting the growth of HeLa cells.

**Scheme 1.**
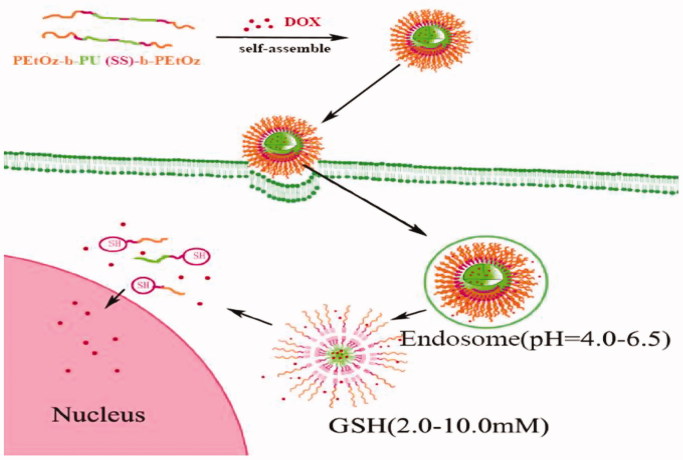
Schematic illustration for self-assembly and dual-responsive drug release of DOX-loaded PEtOz-*b*-PU(SS)-*b*-PEtOz micelles.

Here, we develop a novel shell-detachable micelle with pH- and redox-sensitivity based on an amphiphilic triblock polymer polyoxazoline-block-polyurethane-block-polyoxazoline [PEtOz-*b*-PU(SS)-*b*-PEtOz] for delivery of antitumor drugs to C6 cells (Scheme 1). The triblock amphiphilic polymer PEtOz-*b*-PU(SS)-*b*-PEtOz was easily synthesized from poly(caprolactone) diol containing disulfide bonds in the main chain (PCL-SS-PCL), bis (2-isocyanatoethyl) disulfide (CDI), and polyoxazoline with hydroxyl end groups (PEtOz-OH). PEOz-OH is a pH-sensitive polymer with good biocompatibility that can be protonated at the pH of the endo/lysosome (Li et al., 2015; X. Wang et al., 2011). Thus, PEtOz-*b*-PU(SS)-*b*-PEtOz micelles can quickly escape from endo/lysosomes. The disulfide bonds in the backbone of the PU were formed via the condensation reaction of PCL-SS-PCL and CDI created sensitivity to high levels of GSH in the cytoplasm. These disulfides also affect the degradation speed of the nanoassembly (Yin et al., 2016; Zhang et al., 2018), and we also synthesized another triblock polymer PEtOz-*b*-PU-*b*-PEtOz from PCL-SS-PCL, 1,6-diisocyanatohexane (HDI) and PEtOz-OH: This system has a similar structure but fewer disulfide bonds in the backbone; it serves as a control system.

## Experimental

### Materials

*ε*-caprolactone (*ε*-CL, 99%) and Stannous octoate [Sn (Oct)_2_] were obtained from Alfa Aesar (Shanghai, China). CDI and HDI were obtained from Energy-Chemical (Shanghai, China). 2-Ethyl-2-oxazoline (EtOz-OH, 99%) were purchased from Alfa Aesar (Shanghai, China), and distilled prior to use. *N,N*-dimethylformamide (DMF), dichloromethane, and toluene was purchased from Sinopharm Chemical Reagent Co. Ltd. (Shanghai, China). 1, 4-dithio-d, l-threitol (DTT) was obtained from Merck (Shanghai, China). Doxorubicin hydrochloride (DOX⋅HCl) was obtained from Beijing ZhongShuo Pharmaceutical Technology Development Co., Ltd. (Beijing, China). While the Roswell Park Memorial Institute medium (RPMI-1640, Thermo Fisher Scientific), fetal bovine serum (FBS, Gibco, Australia), and 96-well plates were obtained from Corning Costar (Shanghai, China). 3-(4, 5-dimethylthiazolyl-2-yl)-2, 5-diphenyltetrazolium bromide (MTT) was purchased from Biosharp (Hefei, China). 4′, 6-diamidino-2-phenylindole (DAPI) was purchased from Roche (Shanghai, China). All other chemicals were used as received.

### Characterization

The ^1^H NMR spectra were recorded on a Bruker 400 MHz (Bruker, Billerica, MA, USA) apparatus using deuterated chloroform (C*D*Cl_3_) as the solvent. The molecular weight and polydispersity of copolymers were determined by a PL GPC 50 instrument (Agilent, Santa Barbara, CA, USA) equipped with Jordi GPC columns (10E4, 2M) following a differential refractive-index detector (PL-RI). The measurements were performed using DMF as the eluent at a flow rate of 1 mL/min at 50 °C and a series of narrow polystyrene standards for the calibration of the columns. The size and zeta-potential of micelles were measured with a Nano-ZS from Malvern Instruments. The morphologies of PEtOz-*b*-PU(SS)-*b*-PEtOz micelles and PEtOz-*b*-PU(SS)-*b*-PEtOz micelles were performed by scanning electron microscope (SEM) SU-8010 (Hitachi, Tokyo, Japan). The cellular uptake and intracellular release behaviors of DOX-loaded micelles were observed by fluorescence microscope (Nikon eclipse Ts2R, Tokyo, Japan). OD values for each well in the assay were measured using a Synergy H4 hybrid multi-well plate reader (BioTek, Winooski, VT, USA) in MTT experiments. The fluorescence of DOX was measured by F-4500 FL Spectrophotometer (Hitachi,Tokyo, Japan) at 298 K.

### Synthesis of PEtOz-*b*-PU(SS)-*b*-PEtOz and PEtOz-*b*-PU-*b*-PEtOz

**Figure 1.**
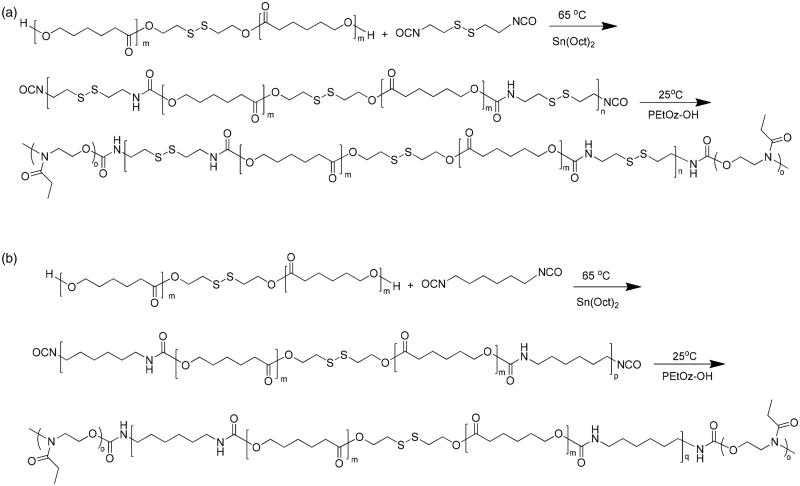
(a) Synthesis route of PEtOz-*b*-PU (SS)-*b*-PEtOz. (b) Synthesis route of PEtOz-*b*-PU-*b*-PEtOz.

The amphiphilic polymer of PEtOz-*b*-PU(SS)-*b*-PEtOz and PEtOz-b-PU-b-PEtOz were synthesized from the PEtOz-OH, PCL-SS-PCL, and CDI or HDI (Figure 1). The PCL-SS-PCL and PEtOz-OH copolymers were synthesized using a similar method as reported (Lee et al., 1999; Kim et al., 2000). PEtOz-*b*-PU(SS)-*b*-PEtOz was synthesized as following routes. First, PCL-SS-PCL (3.00 g, 3.00 mmol) was dissolved in 25 mL DMF under a dry nitrogen atmosphere, then CDI (0.67 g, 3.30 mmol) was added into the DMF solution of PCL-SS-PCL in the presence of 1% Sn (Oct)_2_ (36 mg) at 65 °C with stirring. And this solution was reacted at 65 °C for 24 h. After that, the polymer solution was cooled to room temperature and a solution of PEtOz-OH (3.00 g, 0.60 mmol) in 15 mL DMF was slowly dropped into the above solution under ice-cooling. Then the mixture was stirring for 48 h at 25 °C. The resulting product was obtained by twice precipitation from mixture of ether and methanol (V:V/10:1) to remove the unreacted PEtOz-OH. The polymer was harvested and dried at r.t. under reduced pressure for 24 h to get the final product. Yield: 63.4%.

The copolymer PEtOz-*b*-PU-*b*-PEtOz was synthesized similarly as PEtOz-*b*-PU(SS)-*b*-PEtOz with a yield of 52.1%.

### Preparation and characterization of PEtOz-*b*-PU (SS)-*b*-PEtOz and PEtOz-*b*-PU-*b*-PEtOz micelle

Dialysis method was used to prepare corresponding micelles of polymer PEtOz-*b*-PU(SS)-*b*-PEtOz and PEtOz-*b*-PU-*b*-PEtOz. Briefly, polymer (2 mg) was completely dissolved in 1 mL DMSO. Then, 1.5 mL water was slowly added into the solution under vigorous stirring. Subsequently, the mixture was transferred into a dialysis bag (MWCO 3500 Da) and dialyzed against water for 24 h.

The size and zeta-potential of micelles were measured with a Nano-ZS from Malvern Instruments. The morphologies of PEtOz-*b*-PU(SS)-*b*-PEtOz micelles and PEtOz-*b*-PU-*b*-PEtOz micelles were prepared by dropping 10 uL of micelle (0.1 mg/mL) on monocrystalline silicon, and then dried at room temperature.

The critical micelle concentration (CMC) of PEtOz-*b*-PU (SS)-*b*-PEtOz micelles was determined by fluorescence spectroscopy using pyrene as fluorescent probe. The concentration of polymer was varied from 1.0 × 10^−4^ to 0.1 g/L and the final pyrene concentration was fixed at 0.6 μM. The fluorescence of micelle solution was measured by fluorescence spectrophotometer at wavelength range from 340 to 500 nm with the excitation wavelength of 330 nm.

### Stability of PEtOz-*b*-PU(SS)-*b*-PEtOz micelle in different condition

The stability of PEtOz-*b*-PU(SS)-*b*-PEtOz micelles was determined by the dynamic light scattering (DLS) measurement at 25 °C. We assessed the degradation behavior of PEtOz-*b*-PU(SS)-*b*-PEtOz micelles by monitoring the size change of micelle under reductive and acidic environment (mimic intracellular environment). Briefly, micellar solution in acetate buffer with DTT (10 mM, pH 5.0) under N_2_ atmosphere. The solution was stirred by shaker at 37 °C for 24 h. During this period, we measured the size change of PEtOz-*b*-PU(SS)-*b*-PEtOz micelle. In this experiment, we used micelle in different media, such as phosphate buffer (PB; 10 mM, pH 7.4), PB (10 mM DTT, pH 7.4), or acetate buffer (10 mM, pH 5.0) as control.

### Drug loading content (DLC) and drug loading efficiency (DLE) of DOX

The DOX-loaded PU micelles were prepared by dialysis method. Before loading DOX to the PU micelle, DOX·HCl (2 mg, 0.0034 mmol) was taken off HCl by triethylamine (TEA, 0.99 mL, 6.8 mmol) in DMSO (0.4 mL) overnight in the dark. Then, PEtOz-*b*-PU(SS)-*b*-PEtOz or PEtOz-*b*-PU-*b*-PEtOz (5 mg) and DOX (Theoretical DLC = 10, 20 wt.%) was completely dissolved in 2 mL DMSO. Then, 3 mL of water was slowly added into the resulting solution under vigorous stirring in the dark. Subsequently, the micelle solution was transferred to the dialysis bag (MWCO, 3500 Da) and dialyzed against distilled water for 24 h to remove the unloaded DOX and DMSO. DLC was determined by fluorescence measurement (excitation at 480 nm). For determination of DLC, DOX-loaded micelles were lyophilized and dissolved in DMSO and analyzed with fluorescence spectroscopy, wherein calibration curve was obtained with DOX/DMSO solutions with different DOX concentration. DLC and DLE were calculated according to the following formula.

$$\text{DLC}\;\;(\text{wt}.\%)=(\frac{\text{weight}\;\;\text{of}\;\;\text{loaded}\;\;\text{drug}}{\text{total}\;\;\text{weight}\;\;\text{of}\;\;\text{loaded}\;\;\text{drug}\;\;\text{and}\;\;\text{polymer}})\times 100\%$$

$$\text{DLE}\;\;(\%)=(\frac{\text{weight}\;\;\text{of}\;\;\text{loaded}\;\;\text{drug}}{\text{weight}\;\;\text{of}\;\;\text{drug}\text{in}\;\;\text{feed}})\times 100\%$$

### *In vitro* release of DOX

The drug release behaviors of DOX-loaded micelles were investigated in a dialysis bag (MWCO 12,000 Da) which was stirred by shaker at 37 °C. The DOX-loaded micelle was released in four different media, that is, acetate buffer (10 mM, pH 5.0) with 10 mM DTT, acetate buffer (10 mM, pH 5.0), PB (10 mM, pH 7.4) with 10 mM DTT or PB (10 mM, pH 7.4). In order to acquire the sink conditions, drug release studies were performed at a low DLC of 2.5 wt. % and with 0.5 mL of micelle against 25 mL dialysis of appropriate medium. At desired time intervals, 9.0 mL of the release media was taken out and refreshed with an equal volume of fresh medium. The amount of DOX released was measured by fluorescence measurements (excitation at 488 nm, emission at 560 nm).

### Cell viability assay

The *in vitro* cytotoxicity of PEtOz-*b*-PU(SS)-*b*-PEtOz and PEtOz-*b*-PU-*b*-PEtOz micelles were evaluated by MTT assay using C6 cells. The C6 cells were plated in a 96-well plate (5 × 10^3^ cells/well) in RPMI 1640 media supplemented with 10% FBS, 1% l-glutamine, antibiotics penicillin (100 IU/mL) and streptomycin (100 μg/mL). After 24 h, the medium was removed and replaced by 80 μL of fresh medium and 20 μL of blank or drug-loaded PU micelle was added to make final micelle concentrations of 0.1, 0.2, 0.4, 0.8, and 1.0 mg/mL. Then we incubated the cell at 37 °C with 5% CO_2_ for 48 h. At last, the medium in 96-well plate was replaced by 100 μL of fresh medium and 10 μL of MTT solution (5 mg/mL) was added. After 4 h, we aspirated medium and dissolved MTT-formazan in 150 μL of DMSO. The relative cell viability (%) was determined by comparing the absorbance at 490 nm with control wells.

The *in vitro* cytotoxicity of drug loaded PEtOz-*b*-PU(SS)-*b*-PEtOz and PEtOz-*b*-PU-*b*-PEtOz micelles were evaluated by the similar method as PEtOz-*b*-PU(SS)-*b*-PEtOz and PEtOz-*b*-PU-*b*-PEtOz micelles. And the final concentration of DOX in the well was varied from 0.01 μg/mL to 40 μg/mL.

### Cellular uptake and intracellular release

The cellular uptake of micelle was observed by fluorescent inverted microscope (Nikon eclipse Ts2R) in C6 cells. DOX (red fluorescence) was loaded by PU micelles. The red fluorescence of DOX could label the nanocarriers and track the internalization and intracellular localization of DOX in C6 cells. The nuclei of C6 cells were stained by DAPI, which presented blue fluorescence to showed localization of nuclei. Briefly, C6 cells were plated in a confocal dish (8 × 10^4^ cells/well) in 1 mL RPMI 1640 media supplemented with 10% FBS, 1% l-glutamine and antibiotics penicillin (100 IU/mL) and streptomycin (100 μg/mL) for 24 h. The media were aspirated and replaced by 1 mL of fresh medium. Fifty microliters of DOX-loaded micelle or free DOX·HCl (10.0 μg/mL) was added. The cells were incubated at 37 °C for 2 h or 4 h with DOX-loaded micelle or free DOX·HCl in a humidified 5% CO_2_ atmosphere. The culture medium was removed and the cells were washed three times with phosphate buffered saline (PBS, pH 7.4, 10 mM). The cells were fixed with 4% paraformaldehyde for 20 min and washed three times with PBS. Then cells were stained with DAPI for 20 min and washed with PBS for three times. At last, we used fluorescent inverted microscope (Nikon eclipse Ts2R) to obtain fluorescence images of cells.

## Results and discussion

### Characterization of PU copolymers

PEtOz-*b*-PU(SS)-*b*-PEtOz and PEtOz-*b*-PU-*b*-PEtOz were conveniently synthesized via the condensation reaction of poly (ε-caprolactone) diol (PCL-SS-PCL) and diisocyanate (CDI or HDI), which was subsequently reacted with PEtOz-OH. Finally, we washed and repeated precipitation in the mixture of ether and methanol (V:V/10:1) to remove the unreacted PEtOz-OH. The molecular weight of the polymers was calculated via ^1^H NMR. The GPC analyses showed that PEtOz-*b*-PU(SS)-*b*-PEtOz had a unimodal distribution with a moderate PDI of 1.19 and *Mn* value was 2.9 kg/mol (Table S1). Similar as PEtOz-*b*-PU(SS)-*b*-PEtOz, PEtOz-*b*-PU-*b*-PEtOz also had a narrower distribution with a PDI of 1.17 and a *Mn* value was 2.4 kg/mol (Table S1).

**Figure 2.**
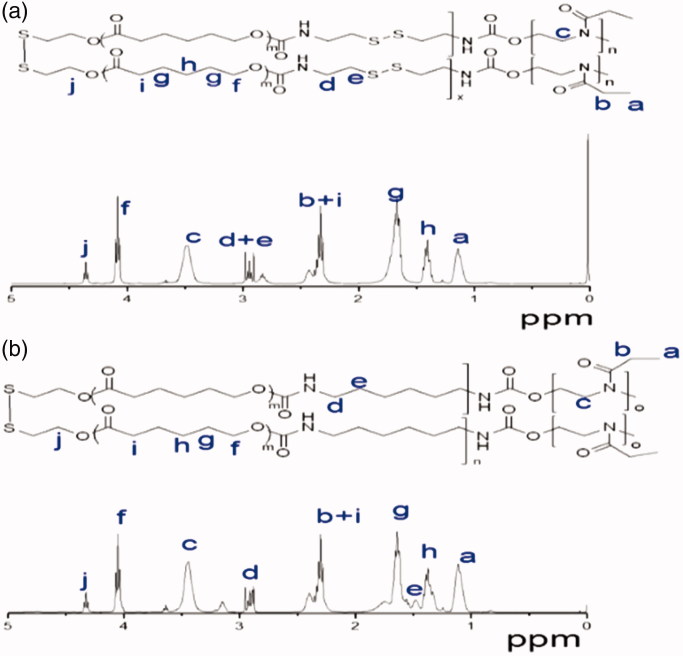
(a) ^1^H NMR spectrum (400 MHz, C*D*Cl_3_) of PEtOz-*b*-PU (SS)-*b*-PEtOz copolymer. (b) ^1^H NMR spectrum (400 MHz, C*D*Cl_3_) of PEtOz-*b*-PU-*b*-PEtOz copolymer.

The ^1^HNMR spectrum also showed that besides signals attribute to PEtOz [1.111 (s), 2.368 (d), 3.444 (s)] and PCL [1.383 (s), 1.674 (s), 2.368 (d), 4.355 (t)], there were also resonances from CDI at 2.829 (s) and 2.905–2.978 (m) (Figure 2(a)). These results suggest successful synthesis of amphiphilic polymer of PEtOz-*b*-PU(SS)-*b*-PEtOz. The ^1^H NMR spectra of PEtOz-*b*-PU-*b*-PEtOz showed signals attributable to PEtOz [1.111 (s), 2.368 (d), 3.444 (s)] and PCL [1.383 (s), 1.674 (s), 2.368 (d), 4.327 (t)] as well as resonances attributable to HDI at 3.166 (s), 2.905–2.978 (m) (Figure 2(b)). End group analysis of ^1^H NMR showed that the *Mn* value of PEtOz-*b*-PU(SS)-*b*-PEtOz is 21.8 kg/mol (Table S1). The difference between *Mn* of GPC and *Mn* of ^1^H NMR is due to the fact that the PUs we synthesized have an obviously different structure from the polystyrenes used as GPC calibration samples (Figure S1).

### Characterization of PU micelles

We used DLS and SEM to monitor the size and surface charge of the PU micelles. Both micelles showed modest negative surface charges (−32.3 mV for PEtOz-*b*-PU-*b*-PEtOz and −31.8 mV for PEtOz-*b*-PU(SS)-*b*-PEtOz, respectively; Table 1). The modest negative surface charges of these micelles facilitate longer blood circulation *in vivo*. The PU micelles had lower average diameters of approximate 152.1 nm for PEtOz-*b*-PU-*b*-PEtOz and 109.9 nm for PEtOz-*b*-PU(SS)-*b*-PEtOz, respectively (Figure S2(a)). Smaller size of micelle was observed in SEM images (Figure S2(b,c)). The size of the micelle in SEM is less than that of DLS because the micelle shrinked after drying, while the DLS measured in the aqueous solution (Wang et al., 2011; Li et al., 2017). The smaller size of the PU micelles means that these micelles could be easily uptaken by cells. The CMC of these micelles was determined via fluorescence spectroscopy using pyrene as a fluorescence probe. The concentration of micelle varied from 1.0 × 10^−4^ to 0.1 g/L, and the concentration of pyrene was fixed at 0.6 μM. The fluorescence spectra of the micelles were measured at an excitation wavelength of 330 nm. The CMC was obtained as the cross-point by extrapolating the intensity ratio I_372_/I_383_ (I_1_/I_3_) at low and high concentration regions (Figure S3). We then got a lower CMC of 2.14 mg/L from the micelles based on PEtOz-*b*-PU-*b-*PEtOz, and 1.99 mg/L from micelles based on PEtOz-*b*-PU(SS)-*b*-PEtOz in PB (10 mM, pH 7.4) (Table 1). These lower CMC values indicate that these polymers can form micelles at very low concentrations. Therefore, the micelles are stable even upon dilution in body fluids after *in vivo* administration.

**Table 1.** Characterization of PEtOz-*b*-PU-*b*-PEtOz micelles and PEtOz-*b*-PU(SS)-*b*-PEtOz micelles.

Copolymer	Size (nm)^a^	PDI^a^	CMC (mg/L)^b^	Zata (mV)^a^
PEtOz-*b*-PU-*b*-PEtOz	152.1 ± 2.3	0.19	2.14	−32.3
PEtOz-*b*-PU(SS)-*b*-PEtOz	109.9 ± 1.6	0.08	1.99	−31.8

a Determined by DLS at a concentration of 0.25 mg/mL at 25 °C in water.

b Determined by fluorescence measurement using pyrene as a fluorescence probe (pyrene final concentration is 0.6 μM).

The stability of PEtOz-*b*-PU(SS)*-b*-PEtOz micelles in reductive and/or acidic condition was also studied by DLS in PB (10 mM, pH 7.4) at 37 °C. Notably, the size of PEtOz-*b*-PU(SS)-*b*-PEtOz micelles did not change much within 24 h in PB (10 mM, pH 7.4) indicating that PEtOz-*b-*PU(SS)-*b-*PEtOz micelles have adequate stability in body fluids environment. This guarantees the stability of drug-loaded micelles in blood; however, the size of PEtOz-*b*-PU(SS)-*b*-PEtOz micelles obviously changed under reducing conditions (10 mM DTT) or acidic environment (pH 5.0) (Figure S4(a,b)). As we can see in Figure S4, the PEtOz-*b*-PU(SS)-*b*-PEtOz micelles swelled to over 500 nm in 4 h under a reducing environment (10 mM DTT). Figure S4(c) shows that PEtOz-*b-*PU (SS)-*b*-PEtOz micelles swelled to over 400 nm in 4 h and split from a single peak into multiple peaks in a few hours under reducing and acidic environments (10 mM DTT, pH 5.0). In contrast, the PEtOz-*b*-PU-*b*-PEtOz micelles did not change as much as PEtOz-*b*-PU-*b*-PEtOz micelles under the same condition (Figure S4(d)). These results confirm that PEtOz-*b*-PU(SS)-*b*-PEtOz micelles could respond to either reductive or acidic intracellular conditions more quickly than PEtOz-*b*-PU-*b*-PEtOz micelles. This is because the PEtOz-*b*-PU(SS)-*b*-PEtOz micelles could not only swell in acidic conditions but also could detach from the outer shell of PEtOz in reducing environments. These findings also verified that the PEtOz-*b*-PU (SS)-*b*-PEtOz micelle and PEtOz-*b*-PU-*b*-PEtOz micelle can degrade rapidly in the intracellular environment of cancer cells.

### *In vitro* release of DOX

DOX was selected as a model hydrophobic anticancer drug to evaluate the drug-loading efficiency of the obtained PU micelles. The DLC and DLE were calculated according to the standard DOX curve. Table S2 shows the drug-loading efficiency of PEtOz-*b*-PU(SS)-*b*-PEtOz micelles decreased from 52% to 43% with the drug-loading capacity increasing from 5.2% to 8.7%. In contrast to blank micelles of PEtOz-*b*-PU(SS)-*b*-PEtOz, the size of the DOX-loaded PEtOz-*b*-PU(SS)-*b*-PEtOz micelles increased by 14–90 nm, and the distribution became slightly wider. The drug-loaded micelles of this size can be easily phagocytosed by cancer cells to improve transport of DOX into cancer cells.

**Figure 3.**
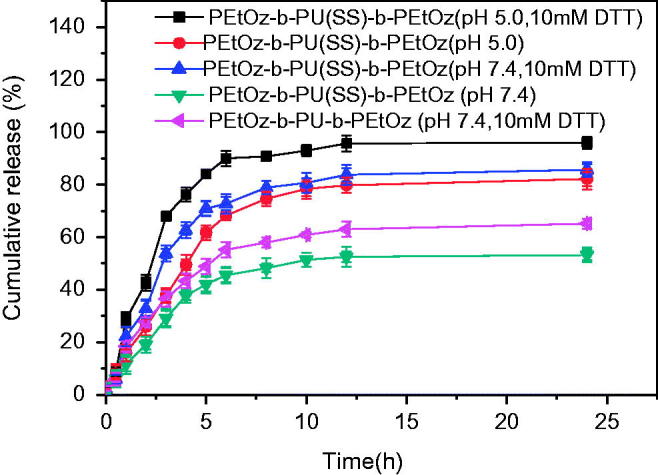
*In vitro* DOX release profile of DOX-loaded PEtOz-*b*-PU(SS)-*b*-PEtOz micelles at pH 7.4 and 5.0 in the presence and absence of 10 mM DTT at 37 °C for 24 h. DOX-loaded PEtOz-*b*-PU-*b*-PEtOz micelles was used as control. Error bars represent standard deviation (SD) for *n* = 3.

The drug release behavior of the DOX-loaded PU micelles was investigated at 37 °C in PB (pH 7.4) or acetate buffer (pH 5.0) with or without 10 mM DTT. Figure 3 shows that 76% DOX was quickly released from DOX-loaded PEtOz-*b*-PU(SS)-*b*-PEtOz micelles in acetate buffer (pH 5.0, 10 mM DTT) within 4 h, and 95% of DOX was released in 24 h. However, in acetate buffer (pH 5.0) without DTT, only 49% of the drug was released within 4 h and 78% of DOX was released in 24 h. At 10 mM DTT with PB (pH 7.4), 62% was released in 4 h and 83% DOX in 24 h. In addition, in PB (pH 7.4) without DTT, DOX-loaded PEtOz-*b*-PU(SS)-*b*-PEtOz micelles only released 38% DOX in 4 h and 52% DOX within 24 h. For DOX-loaded PEtOz-*b*-PU-*b*-PEtOz micelles, only 65% of the drug was released at 10 mM DTT with PB (pH 7.4) within 24 h. We also noticed that the burst release of DOX-loaded PEtOz-*b*-PU(SS)-*b*-PEtOz micelles was nearly 40%. The possible reason may be that, hydrophilic shell of our assembled micelles consists of PEtOz, the PEtOz shell may has some hydrophobicity under the physiological conditions for its p*K*_a_ was lower than 7.4. It might result in some drugs adsorption to the PEtOz shell during drug loading. Then during the swelling process of the PEtOz shell, these drugs are released. These reasons led to the drug premature release for PEtOz-*b*-PU(SS)-*b*-PEtOz drug-loaded micelles. We also observed the similar phenomenon in following references, in these references, micelles were prepared from polymers with similar structure of PEtOz hydrophilic segment (Gao et al., 2015; Li et al., 2015).

Versus prior work, the cumulative drug release of the DOX-loaded PEtOz-*b*-PU(SS)-*b*-PEtOz micelles was increased by ca. 20% in 24 h at pH 5.0 in the presence of DTT. The enhanced DOX release is due to the structure of the PU micelle: The disulfide bonds of the PU micelles can rapidly break under reducing conditions, and PEtOz can quickly swell under acidic environments. These features give PEtOz-*b*-PU(SS)-*b*-PEtOz micelles excellent drug release into cancer cells.

### Cell viability assay

**Figure 4.**
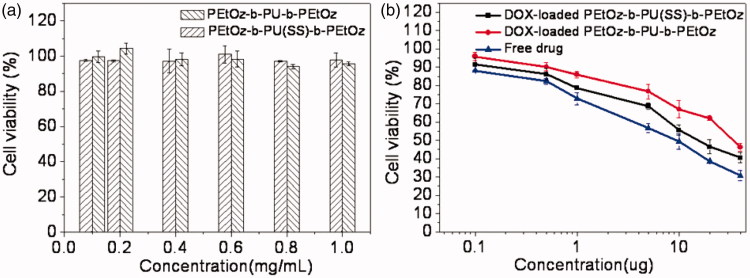
(a) Cytotoxicity of PEtOz-*b*-PU (SS)-*b*-PEtOz and PEtOz-*b*-PU-*b*-PEtOz micelles, against C6 cells after 24 h incubation using MTT assay. All data represent mean ± SD (*n* = 6). (b) Antitumor activities of DOX-loaded PEtOz-*b*-PU (SS)-*b*-PEtOz micelles in C6 cells. DOX-loaded PEtOz-*b*-PU-*b*-PEtOz micelles were used as controls. The cells were treated with DOX-loaded micelles or free DOX for 48 h. Data are presented as mean ± SD (*n* = 6).

The *in vitro* cytotoxicity of PEtOz-*b*-PU(SS)-*b*-PEtOz and PEtOz-*b*-PU-*b*-PEtOz micelles were evaluated with an MTT assay using C6 cells. The results revealed that PEtOz-*b*-PU(SS)-*b*-PEtOz and PEtOz-*b*-PU-*b*-PEtOz micelles had no significant toxic effect on C6 cells even at a high concentration of 1.0 mg/mL for 48 h (Figure 4(a)). This phenomenon suggested that PEtOz-*b*-PU(SS)-*b*-PEtOz and PEtOz-*b*-PU-*b*-PEtOz micelles have excellent biocompatibility. In contrast, DOX-loaded PEtOz-*b*-PU(SS)-*b*-PEtOz and PEtOz-*b*-PU-*b*-PEtOz micelles had toxicity on C6 cells. The half-maximal inhibitory concentration (IC50) of DOX-loaded PEtOz-*b*-PU(SS)-*b*-PEtOz micelles was 18.1 μg DOX equiv./mL in C6 cells; the IC50 of DOX-loaded PEtOz-*b*-PU-*b*-PEtOz micelles was 39.7 μg DOX equiv./mL in C6 cells (Figure 4(b)). However, the IC50 of free DOX•HCl was only 3.5 μg/mL. The IC50 value of DOX-loaded micelles was higher than free DOX•HCl because of the different drug efficacy of micelles and inefficient cellular uptake.

DOX-loaded PEtOz-*b*-PU (SS)-*b*-PEtOz micelles were more toxic than DOX-loaded PEtOz-*b*-PU-*b*-PEtOz micelles against C6 cells. This is because the DOX-loaded PEtOz-*b*-PU(SS)-*b*-PEtOz micelle could release the loaded drug more thoroughly than the DOX-loaded PEtOz-*b*-PU-*b*-PEtOz micelle – these micelles were prepared from two amphiphilic polymers with different numbers and locations of disulfide bonds, which were cleaved in an intracellular reducing environment. The *in vitro* cytotoxicity also confirms that the PEtOz-*b*-PU(SS)-*b*-PEtOz micelle could deliver more drug to tumor cells. The PEtOz-*b*-PU(SS)-*b*-PEtOz has more disulfide bonds than PEtOz-*b*-PU-*b*-PEtOz, but PEtOz-*b*-PU(SS)-*b*-PEtOz has disulfide bonds between the hydrophilic segment and hydrophobic segment. This disulfide increases release. Such micelles could release loaded drug more rapidly and completely. In contrast, the PEtOz-*b*-PU-*b*-PEtOz micelle with disulfide bonds only in the hydrophobic segment had a weaker ability to release encapsulated drug and thus displayed weaker toxicity to tumor cells.

### Cellular uptake and intracellular release

**Figure 5.**
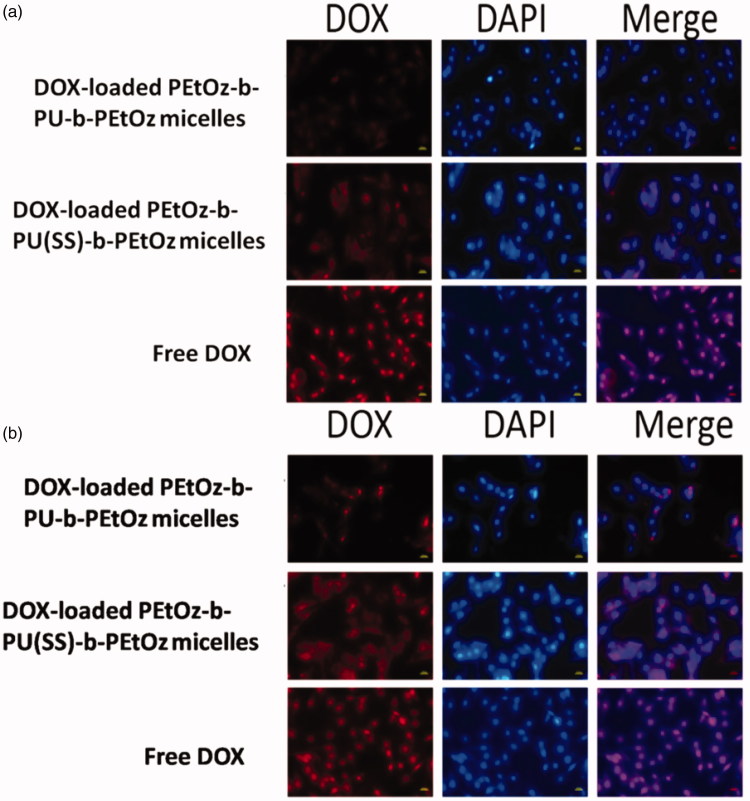
(a) Fluorescence microscope images of C6 cells incubated with DOX-loaded PEtOz-*b*-PU(SS)-*b*-PEtOz micelles, DOX-loaded PEtOz-*b*-PU-*b*-PEtOz micelles and free DOX (10 µg/mL) in 2 h. (b) Fluorescence microscope images of C6 cells incubated with DOX-loaded PEtOz-*b*-PU(SS)-*b-*PEtOz micelles, DOX-loaded PEtOz-*b*-PU-*b*-PEtOz micelles and free DOX (10 µg/mL) in 4 h. For each panel, images from left to right show DOX fluorescence in cells (red),cell nuclei stained by DAPI (blue) and overlays of two images. The scale bars correspond to 20 µm in all the images.

The cellular uptake of the drug-loaded PEtOz-*b*-PU(SS)-*b*-PEtOz and PEtOz-*b*-PU-*b*-PEtOz micelles were observed with fluorescent microscopy (Nikon eclipse Ts2R) in C6 cells (Figure 5). The cells incubated with DOX-loaded PEtOz-*b*-PU(SS)-*b*-PEtOz micelles showed DOX fluorescence in the cytoplasm and nucleus after 2 h or 4 h of incubation. In contrast, cells with DOX-loaded PEtOz-*b*-PU-*b*-PEtOz micelles had weaker DOX fluorescence. This further confirms that DOX-loaded PEtOz-*b*-PU(SS)-*b*-PEtOz micelles release the loaded drug more thoroughly than DOX-loaded PEtOz-*b*-PU-*b*-PEtOz micelles. The system with disulfide bonds between hydrophilic and hydrophobic segments can form shell-detachable micelles that easily break down in the intracellular reductive environment resulting in more complete drug release. Due to inefficient cellular uptake at predetermined incubation times, we observed lower DOX fluorescence for cells cultured with DOX-loaded micelles than those with free DOX. Therefore, we concluded that DOX-loaded PEtOz-*b*-PU(SS)-*b*-PEtOz micelles have excellent and efficient ability to release DOX via pH and redox effects.

## Conclusions

We developed pH- and redox-responsive micelles based on PU. These materials have a different number of disulfide bonds that efficiently deliver and release DOX into C6 cells resulting in superior antitumor activity. The polymer PEtOz-*b*-PU(SS)-*b*-PEtOz with disulfide bonds between the hydrophilic and hydrophobic segments had better drug release capabilities. The *in vitro* cytotoxicity of PEtOz-*b*-PU(SS)-*b*-PEtOz micelle showed good biocompatibility, which is important for biomedical applications. The micelles exhibit fast reduction and pH-triggered drug release, and DOX-loaded PEtOz-*b*-PU(SS)-*b*-PEtOz micelles significantly enhance DOX cytotoxicity against C6 cells. These dual-responsive biodegradable micelles are promising for glioma treatment, which will be evaluated in future work.

## Supplementary Material

Supporting_Information_-revised_20190202.docx
