# Supplementary material for: pH and reduction dual-responsive micelles based on novel polyurethanes with detachable poly(2-ethyl-2-oxazoline) shell for controlled release of doxorubicin
Source: Drug Deliv. 2019 Mar 21;26(1):300–8. doi: 10.1080/10717544.2019.1580323 (PMC6442156; doi:10.1080/10717544.2019.1580323)
Supplement: Supporting_Information_-revised_20190202.docx [file IDRD_A_1580323_SM4363.docx]

(Supporting Information)

**pH and Reduction Dual-Responsive Micelles Based on Novel Polyurethanes with Detachable Poly(2-ethyl-2-oxazoline) Shell for Controlled Release of Doxorubicin**

Leran Bu^a^, Hena Zhang^a^, Kang Xu^a^, Baixiang Du^a^, Caihong Zhu^b^*, Yuling Li^a^*

1. School of Chemistry & Materials Science, Jiangsu Normal University, Xuzhou 221116, China
2. Orthopaedic Institute, Medical College, Soochow University, Suzhou 215007, China

**
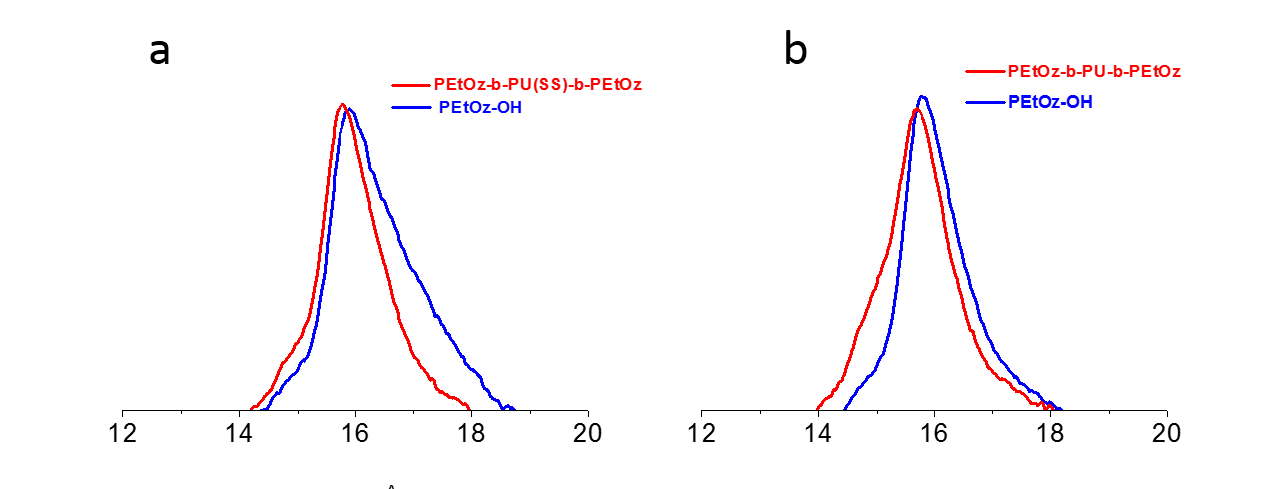
**

**Figure S1.** a) DMF GPC traces of PEtOz-OH and PEtOz-*b*-PU (SS)-*b*-PEtOz copolymer. b) DMF GPC traces of PEtOz-OH and PEtOz-*b*-PU-*b*-PEtOz copolymer.


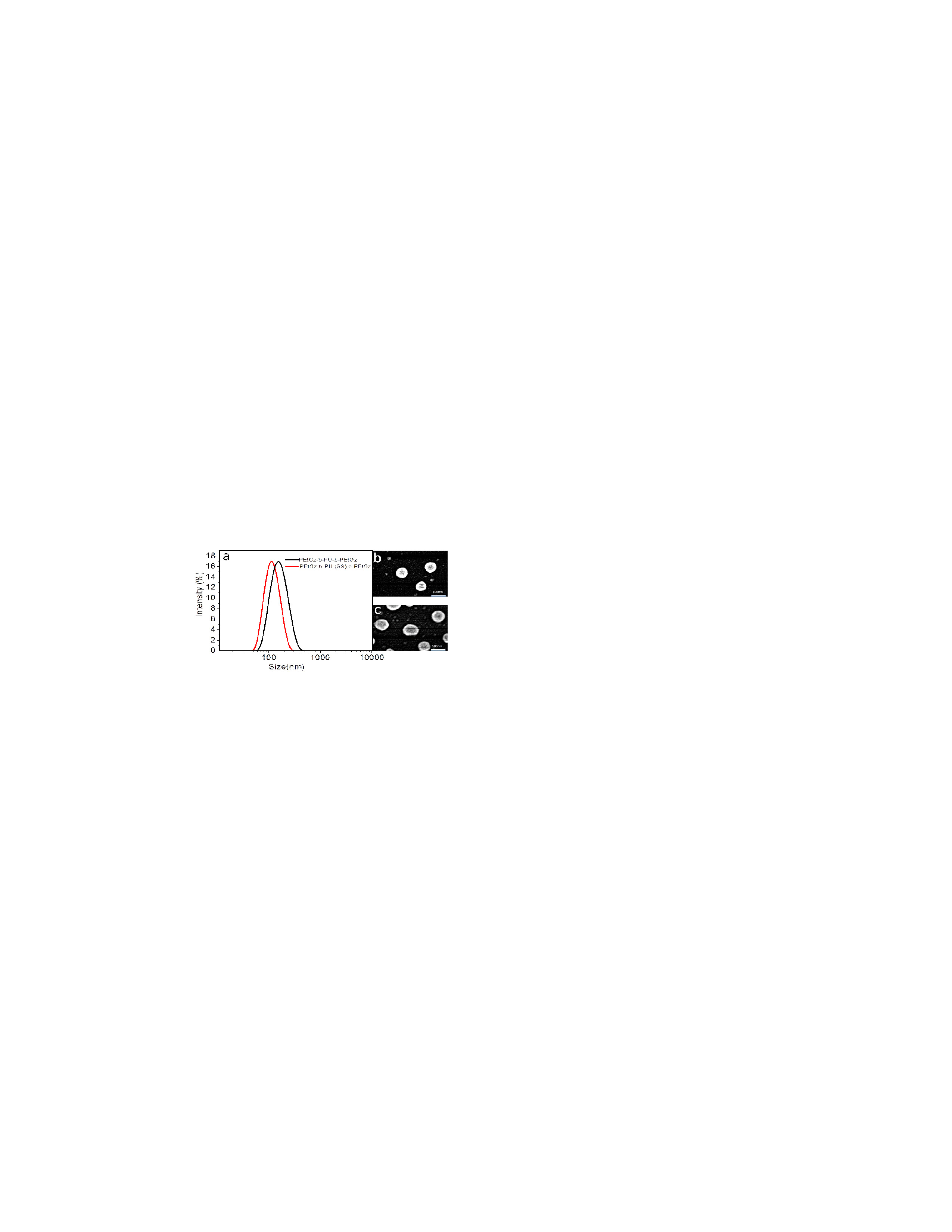


**Figure S2.** a) The size distribution of PEtOz-b-PU-b-PEtOz micelles and PEtOz-b-PU (SS)-b-PEtOz micelles. b) SEM image of PEtOz-b-PU (SS)-b-PEtOz micelles. c) SEM image of PEtOz-b-PU-b-PEtOz micelles.


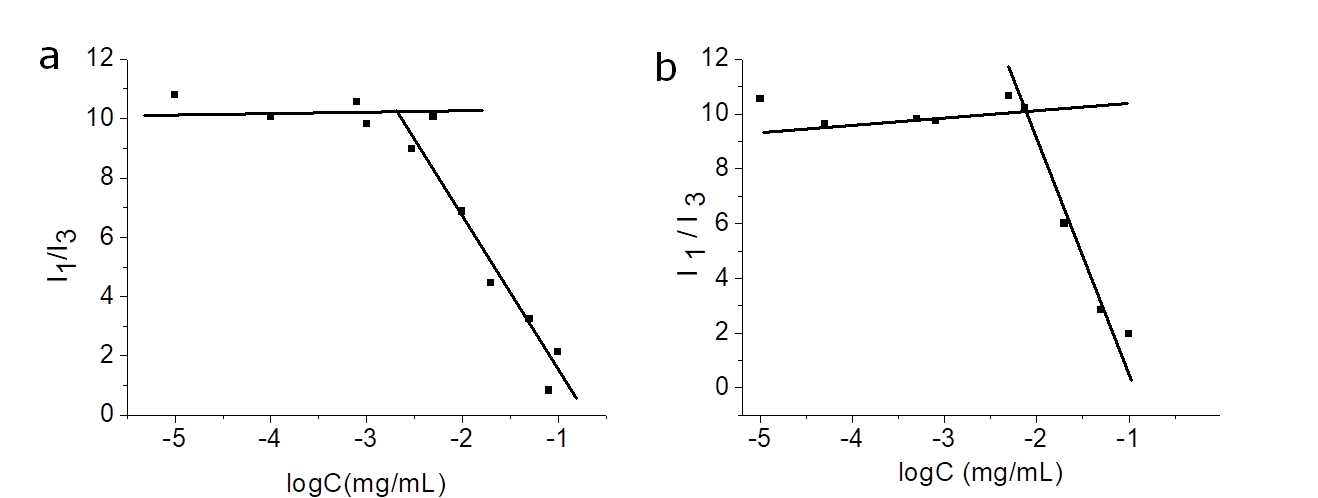


**Figure S3.** The fluorescence intensity ratio I_1_/I_3_ of pyrene as a function of PEtOz-*b*-PU (SS)-*b*-PEtOz micelles concentration (a: PEtOz-*b*-PU-*b*-PEtOz micelles, b: PEtOz-*b*-PU (SS)-*b*-PEtOz micelles).

*^
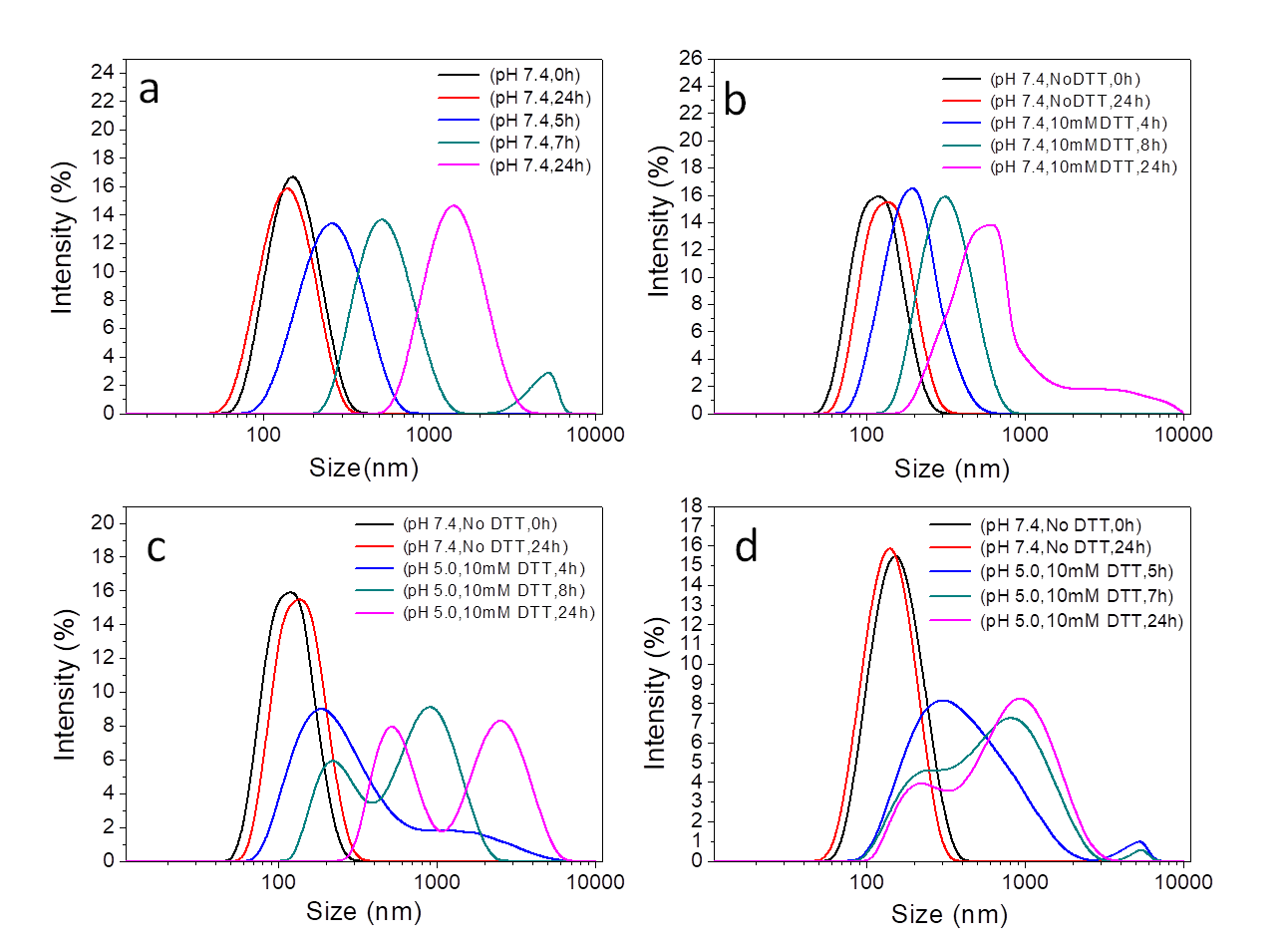
^*

**Figure S4.** Particle size distribution of PEtOz-*b*-PU (SS)-*b*-PEtOz micelles at pH 5.0 or pH 7.4. b) Particle size distribution of PEtOz-*b*-PU (SS)-*b*-PEtOz micelles at pH 7.4 with 10 mM DTT and without 10mM DTT. c) Particle size distribution of PEtOz-*b*-PU(SS)-*b*-PEtOz micelles at pH 5.0 or pH 7.4 with/ without 10mM DTT. d) Particle size distribution of PEtOz-*b*-PU-*b*-PEtOz micelles at at pH 5.0 or pH 7.4 with / without 10mM DTT.

**Figure S5.** DMF GPC traces of PCL-SS-PCL polymer.

**Figure S6.** ^1^H NMR spectrum (400 MHz, C*D*Cl_3_) of PCL-SS-PCL polymer.

**Figure S7.** ^1^H NMR spectrum (400 MHz, C*D*Cl_3_) of PEtOz-OH polymer.

**Table S1.** Characteristics of polymers of PEtOz-OH, PEtOz-*b*-PU(SS)-*b*-PEtOz and PEtOz-*b*-PU-*b*-PEtOz

| Copolymer | *M_n_*(kg mol ^-1^) | | | *M_w_*/*M_n_*^[b]^ |
| --- | --- | --- | --- | --- |
|  | Design | ^1^H NMR^[a]^ | GPC^[b]^ |  |
| PEtOz-OH | 5 | 3.8 | 2.2 | 1.10 |
| PEtOz-*b*-PU(SS)-*b*-PEtOz | 30 | 21.8 | 2.9 | 1.19 |
| PEtOz-b-PU-*b*-PEtOz | 30 | 17.6 | 2.4 | 1.17 |

[a] Calculated from 1H NMR；

[b] Using DMF as mobile phase, polystyrene as a standard, measured by GPC.

**Table S2.** Characteristics of DOX-loaded PEtOz-*b*-PU(SS)-*b*-PEtOz z micelles

| Sample | Micelle | | | | |
| --- | --- | --- | --- | --- | --- |
|  | Theoretical drug loading  wt. % | DLC ^[a]^  wt. % | DLE ^[b]^  % | Size ^[c]^  nm | PDI^[c]^ |
| PEtOz-*b*-PU(SS)-*b*-PEtOz | 10 | 5.23 | 52.37 | 124.4±0.6 | 0.211 |
|  | 20 | 8.71 | 43.58 | 196.0 ±2.1 | 0.217 |

^[a[ ,[b]^ Determined by fluorescence measurement

^[c]^ Determined by DLS at a concentration of 0.25 mg/mL at 25 °C in water.
